# Single Machine Scheduling and Due Date Assignment with Past-Sequence-Dependent Setup Time and Position-Dependent Processing Time

**DOI:** 10.1155/2014/620150

**Published:** 2014-08-27

**Authors:** Chuan-Li Zhao, Chou-Jung Hsu, Hua-Feng Hsu

**Affiliations:** ^1^College of Mathematics and Systems Sciences, Shenyang Normal University, Shenyang, Liaoning 110034, China; ^2^Department of Industrial Management, Nan Kai University of Technology, Nantou 542, Taiwan

## Abstract

This paper considers single machine scheduling and due date assignment with setup time. The setup time is proportional to the length of the already processed jobs; that is, the setup time is past-sequence-dependent (p-s-d). It is assumed that a job's processing time depends on its position in a sequence. The objective functions include total earliness, the weighted number of tardy jobs, and the cost of due date assignment. We analyze these problems with two different due date assignment methods. We first consider the model with job-dependent position effects. For each case, by converting the problem to a series of assignment problems, we proved that the problems can be solved in *O*(*n* ^4^) time. For the model with job-independent position effects, we proved that the problems can be solved in *O*(*n* ^3^) time by providing a dynamic programming algorithm.

## 1. Introduction

In many realistic scheduling environments, a job's processing time may be depending on its position in the sequence [1]. Two well-known special cases of this stream of research are (i)* positional deterioration (aging effect)*, where the processing time of a job increases as a function of its position in a processing sequence and (ii)* learning effect*, where the processing time of a job decreases as a function of its position in a processing sequence. Biskup [2] and Cheng and Wang [3] independently introduced the learning concept to scheduling research. Other studies include Mosheiov and Sidney [4], Mosheiov [5, 6], Wu et al. [7, 8], and Yin et al. [9, 10]. Biskup [11] presented an updated survey of the results on scheduling problems with the learning effect. Mosheiov [6] first mentioned the* aging effect *in scheduling research. Other studies include Mosheiov [12], Kuo and Yang [13], Janiak and Rudek [14], Zhao and Tang [15], and Rustogi and Strusevich [16], among others. Moreover, some studies consider scheduling problems with general position-dependent processing time. Mosheiov [17] considered a scheduling problem with general position-dependent processing time. The polynomial algorithm is derived for makespan minimization on an m-machine proportionate flow shop. Zhao et al. [18] studied scheduling and due date assignment problem. They provided a unified model for solving the single machine problems with rejection and position-dependent processing time. Rustogi and Strusevich [19] presented a critical review of the known results for scheduling models with various positional effects.

Koulamas and Kyparisis [20] first introduced a scheduling problem with past-sequence-dependent (p-s-d) setup time. They assumed that the job setup time is proportional to the sum of processing time of all already scheduled jobs. It is proved that the standard single machine scheduling with p-s-d setup time can be solvable in polynomial time when the objectives are the makespan, the total completion time, and the total absolute differences in completion time, respectively. Wang [21] studied the single machine scheduling problems with time-dependent learning effect and p-s-d setup time considerations. He showed that the makespan minimization problem, the total completion time minimization problem, and the sum of the quadratic job completion time minimization problem can be solved in polynomial time, respectively. Yin et al. [22] considered a single machine scheduling model with p-s-d setup time and a general learning effect. They showed that the single machine scheduling problems to minimize the makespan and the sum of the* k*th power of completion time are polynomially solvable under the proposed model. Hsu et al. [23] presented a polynomial-time algorithm for an unrelated parallel machine scheduling problem with setup time and learning effects to minimize the total completion time. Lee [24] proposed a model with the deteriorating jobs, the learning effect, and the p-s-d setup time. He provided the optimal schedules for some single machine problems. Huang et al. [25] considered some single machine scheduling problems with general time-dependent deterioration, position-dependent learning, and p-s-d setup time. They proved that the makespan minimization problem, the total completion time minimization problem, and the sum of the *μ*th power of job completion time minimization problem can be solved by the SPT rule.

Meeting due dates is one of the most important objectives in scheduling (Gordon et al. [26]). In some situations, the tardiness penalties depend on whether the jobs are tardy, rather than how late they are. In these cases, the number of tardy jobs should be minimized (Yin et al. [27]). Kahlbacher and Cheng [28] considered scheduling problems to minimize costs for earliness, due date assignment, and weighted number of tardy jobs. They presented nearly a full classification for the single and multiple machine models. Shabtay and Steiner [29] studied two single machine scheduling problems. The objectives are to minimize the sum of weighted earliness, tardiness, and due date assignment penalties and minimize the weighted number of tardy jobs and due date assignment costs, respectively. They proved that both problems are strongly NP-hard and give polynomial solutions for some important special cases. Koulamas [30] considered the second problem of Shabtay and Steiner [29]. He presented a faster algorithm for a due date assignment problem with tardy jobs. Gordon and Strusevich [31] addressed the problems of single machine scheduling and due date assignment problems in which a job's processing time depends on its position in a processing sequence. The objective functions include the cost of the due dates, the total cost of discarded jobs that cannot be completed by their due dates, and the total earliness of the scheduled jobs. They presented polynomial-time dynamic programming algorithms for solving problems with two due date assignment methods, provided that the processing time of the jobs is positionally deteriorating. Hsu et al. [32] extended part of the objective functions proposed by Gordon and Strusevich [31] to the positional weighted earliness penalty and showed that the problems remain solvable in polynomial time.

## 2. Problem Formulation and Preliminaries

This paper studies the single machine scheduling problems with simultaneous consideration of due date assignment, p-s-d setup time, and position-dependent processing time.

The problem can be described as follows.

A set *N* = {*J*_1_, *J*_2_,…, *J*_*n*_} of *n* jobs has to be scheduled on a single machine. All jobs are available for processing at time zero and preemption is not permitted. Each job *J*_*j*_ has a basic processing time *p*_*j*_. The actual processing time of job *J*_*j*_, if scheduled in position *r* of a sequence, is given by

(1) $$\begin{array}{c} p_{j}^{A}=g\left(j,r\right)p_{j}, \end{array}$$

where *g*(*j*, 1), *g*(*j*, 2),…, *g*(*j*, *n*) represent an array of job-dependent positional factors.

Each job *J*_*j*_ ∈ *N* has to be assigned a due date *d*_*j*_, by which it is desirable to complete that job. Given a schedule, denote the completion time of job *J*_*j*_ by *C*_*j*_. Job *J*_*j*_ is called tardy if *C*_*j*_ > *d*_*j*_, and it is called nontardy if *C*_*j*_ ≤ *d*_*j*_. Let *U*_*j*_ = 1 if job *J*_*j*_ is tardy and let *U*_*j*_ = 0 if job *J*_*j*_ is nontardy. The earliness of *J*_*j*_ is defined as *E*_*j*_ = *d*_*j*_ − *C*_*j*_, provided that *C*_*j*_ ≤ *d*_*j*_. In all problems considered in this paper, the jobs in set *N* have to be split into two subsets denoted by *N*_*E*_ and *N*_*T*_. We refer to the jobs in set *N*_*E*_ as “nontardy,” while the jobs in set *N*_*T*_ are termed “tardy.” A penalty *β*_*j*_ is paid for the tardy job *J*_*j*_ ∈ *N*_*T*_. Given a schedule *π* = [*J*_[*i*]_, *J*_[2]_,…, *J*_[*n*]_], we assumed that the p-s-d setup time of *J*_[*j*]_ is given as Koulamas and Kyparisis [20] did, as follows:

(2) $$\begin{array}{c} s_{\left[j\right]}=\delta \overset{j-1}{\underset{i=1}{\sum }}p_{\left[i\right]}^{A},\;j=2,3,\ldots ,n,\;\;s_{\left[1\right]}=0, \end{array}$$

where *δ* ≥ 0 is a normalizing constant.

The purpose is to determine the optimal due dates and the processing sequence such that the following function is minimized:

(3) $$\begin{array}{c} F\left(d,\pi \right)=\alpha \sum_{J_{j}\in N_{E}}E_{j}+\sum_{J_{j}\in N_{T}}\beta_{j}U_{j}+\varphi \left(d\right), \end{array}$$

where *π* is the sequence of jobs, *α* is the positive unit earliness cost, **d** is the vector of the assigned due dates, and *φ*(**d**) denotes the cost of assigning the due dates that depends on a specific rule chosen for due date assignment. We denote the problem as

(4) $$\begin{array}{c} 1\left|p_{j}^{A}=p_{j}g\left(j,r\right),S_{\text{psd}}\right|\alpha \sum_{J_{j}\in N_{E}}E_{j}+\sum_{J_{j}\in N_{T}}\beta_{j}U_{j}+\varphi \left(d\right). \end{array}$$

Most of the presented results hold for a general positional effect, that is, for any function *g*(*j*, *r*) that depends on both position *r* and job *J*_*j*_. For each individual model, there is a particular rule that defines *g*(*j*, *r*) and explains how exactly the value of *p*_*j*_ changes, for example.

*(i) Job-Dependent Learning Effect (Mosheiov and Sidney [4]).* The actual processing time of a job *J*_*j*_, if scheduled in position *r* of a sequence, is given by

(5) $$\begin{array}{c} p_{j}^{A}=p_{j}r^{a_{j}}, \end{array}$$

where *a*_*j*_ ≤ 0 is a job-dependent learning parameter (include *a*_*j*_ = *a* as a special case, i.e., *p*_*j*_^*A*^ = *p*_*j*_ *r* ^*a*^, Biskup [2]).

*(ii) Job-Dependent Aging Effect (Zhao and Tang [15]).* The actual processing time of a job *j*, if scheduled in position *r* of a sequence, is given by

(6) $$\begin{array}{c} p_{j}^{A}=p_{j}r^{a_{j}}, \end{array}$$

where *a*_*j*_ ≥ 0 is a job-dependent aging parameter (include *a*_*j*_ = *a* as a special case, i.e., *p*_*j*_^*A*^ = *p*_*j*_ *r* ^*a*^, Moshieov [17]).

*(iii) Positional Exponential Deterioration (Wang [33]).* The actual processing time of a job *j*, if scheduled in position *r* of a sequence, is given by

(7) $$\begin{array}{c} p_{j}^{A}=p_{j}a^{r-1}, \end{array}$$

where *a* ≥ 1 is a given positive constant representing a rate of deterioration, which is common for all jobs.

We study our problem with the two most frequently used due date assignment methods.

*(i) The Common Due Date Assignment Method *(usually referred to as* CON*). Where all jobs are assigned the same due date, such that is *d*_*j*_ = *d* for *j* = 1,2,…, *n* and *d* ≥ 0 is a decision variable.

*(ii) The Slack Due Date Assignment Method *(usually referred to as* SLK*). Where all jobs are given an equal flow allowance that reflects equal waiting time (i.e., equal slacks), such that is  *d*_*j*_ = *p*_*j*_^*A*^ + *q* for *j* = 1,2,…, *n* and *q* ≥ 0 is a decision variable.

We first provide some lemmas.

**Lemma 1 (Hardy et al. [34]).**

Let there be two sequences of numbers *x*_*i*_ and *y*_*i*_ (*i* = 1,2,…, *n*). The sum ∑_*i*=1_^*n*^ *x*_*i*_ *y*_*i*_ of products of the corresponding elements is the least if the sequences are monotonically ordered in the opposite sense.

It is not difficult to see that the following property is valid for both the variants of our problem.

**Lemma 2 .**

There exists an optimal schedule in which the following properties hold: (1) all the jobs are processed consecutively without idle time and the first job starts at time 0 for both the variants of the problem; (2) all the nontardy jobs are processed before all the tardy jobs for both the variants of the problem.

## 3. The* CON *Due Date Assignment Method

In the* CON* model, *d*_*j*_ = *d* (*j* = 1,2,…, *n*). We choose *γd* as the cost function *φ*(**d**), where *γ* is a positive constant. Thus, it follows from function (3) that our problem is to minimize the objective function:

(8) $$\begin{array}{c} F\left(d,\pi \right)=\alpha \sum_{J_{j}\in N_{E}}E_{j}+\sum_{J_{j}\in N_{T}}\beta_{j}U_{j}+\gamma d. \end{array}$$

The problem denotes

(9) $$\begin{array}{c} 1\left|p_{j}^{A}=p_{j}g\left(j,r\right),S_{\text{psd}},CON\right|\alpha \sum_{J_{j}\in N_{E}}E_{j}+\alpha \sum_{J_{j}\in N_{T}}\beta_{j}U_{j}+\gamma d. \end{array}$$

Kahlbacher and Cheng [28] provide an *O*(*n* ^4^) time algorithm for the problem 1|*CON*|*α*∑_*J*_*j*_∈*N*_*E*__ *E*_*j*_ + ∑_*J*_*j*_∈*N*_*T*__ *β*_*j*_ *U*_*j*_ + *γd*. In this section, we consider a generalization of the basic model with p-s-d setup times and position-dependent processing times. As a result of Lemma 2, we can restrict our attention to those schedules without idle times and search for the optimal schedule only among the schedules in which one of the jobs is on time.

Let *π* = [*J*_[*i*]_, *J*_[2]_,…, *J*_[*n*]_]; then

(10)  C[1]=g([1],1)p[1],C[2]=C[1]+s[2]+g([2],2)p[2]=g([1],1)p[1]+δg([1],1)p[1]+g([2],2)p[2]=∑k=12[1+(2−k)δ]g([k],k)p[k],⋮C[j]=∑k=1j[1+(j−k)δ]g([k],k)p[k],⋮ C[n]=∑k=1n[1+(n−k)δ]g([k],k)p[k]. ∑j=1nC[j]=∑j=1n(n−j+1)(1+δ(n−j)2)g([j],j)p[j].

Note that we need only to consider the schedule in which all the nontardy jobs are processed before all the tardy jobs.

**Lemma 3 .**

For the problem

(11) $$\begin{array}{c} 1\left|p_{j}^{A}=p_{j}g\left(j,r\right),S_{psd},CON\right|\alpha \sum_{J_{j}\in N_{E}}E_{j}+\sum_{J_{j}\in N_{T}}\beta_{j}U_{j}+\gamma d, \end{array}$$

if the number of jobs in *N*_*E*_ is *h* (denote |*N*_*E*_| = *h*), there exists an optimal schedule *π* = [*J*_[1]_, *J*_[2]_,…, *J*_[*n*]_], such that *d* = *C*_[*h*]_.

**Proof**

Suppose *π* = [*J*_[*i*]_, *J*_[2]_,…, *J*_[*n*]_] is an optimal schedule. Since jobs *J*_[*i*]_, *J*_[2]_,…, *J*_[*h*]_ are nontardy, then *d* ≥ *C*_[*h*]_. Let Δ = *d* − *C*_[*h*]_. Moving the due date Δ units of time to the left such that *d* = *C*_[*h*]_, the objective value will be decreasing (*αj* + *γ*)Δ, which is nonnegative. This means we can find a schedule with *d* = *C*_[*h*]_ that is at least as good as *π*.

As a consequence of Lemma 3, we consider the schedule *π* = [*J*_[*i*]_, *J*_[2]_,…, *J*_[*n*]_] with *d* = *C*_[*h*]_.

Thus,

(12) F(d,π)=α∑Jj∈NEEj+∑Jj∈NTβjUj+γd=α∑j=1hE[j]+∑j=h+1nβ[j]+γd=α∑j=1h[C[h]−C[j]]+∑j=h+1nβ[j]+γC[h]=(αh+γ)(∑j=1h[1+(h−j)δ]g([j],j)p[j]) −α∑j=1h(h−j+1)(1+12δ(h−j))g([j],j)p[j] +∑j=h+1nβ[j]=∑j=1h{α(j−1)+12αδ(h−j)(h+j−1)+[1+δ(h−j)]γ}g([j],j)p[j] +∑j=h+1nβ[j]=∑j=1hwjg([j],j)p[j]+∑j=h+1nβ[j],

where

(13) wj={α(j−1)+12αδ(h−j)(h+j−1)+[1+δ(h−j)]γ}, j=1,2,…,h.

Let *x*_*j*,*r*_ be a binary variable such that *x*_*j*,*r*_ = 1 if job *J*_*j*_ is scheduled in the* r*th position and *x*_*j*,*r*_ = 0; otherwise, *j*, *r* = 1,2,…, *n*. From (12), we can formulate the problem with objective (8) as the following assignment problem* A*1(*h*), which can be solved in *O*(*n* ^3^) time:

(14)  Min⁡  ∑j=1n∑r=1ncj,rxj,rs.t. ∑r=1nxj,r=1, j=1,2,…,n,∑j=1nxj,r=1, r=1,2,…,n,xj,r∈{0,1}, j=1,2,…,n,  r=1,2,…,n,

where

(15) cj,r={wrg(j,r)pjfor  r=1,2,…,hβjfor  r=h+1,  h+2,…,n,wr={α(r−1)+12αδ(h−r)(h+r−1)+[1+δ(h−r)]γ}, r=1,2,…,h.

Note that *c*_*j*,*r*_ is the cost of assigning job *J*_*j*_ (*j* = 1,2,…, *n*) in the* r*th (*r* = 1,2,…, *n*) position in the schedule.

In order to derive the optimal solution, we have to solve the above assignment problem* A*1(*h*) for any *h* = 1,2,…, *n*. We summarize the results of the above analysis and present the following solution algorithm.

**Algorithm 4 .**

* *

*Step 1.* For *h* = 0 (*d* = 0, all the jobs are tardy), calculate *F*(0) = ∑_*j*=1_^*n*^ *β*_*j*_.

*Step 2.* For *h* from 1 to *n*, solve the assignment problem* A*1(*h*) and calculate the corresponding objective value *F*(*h*).

*Step 3.* The optimal value of the function *F* is equal to min⁡{*F*(*h*)∣*h* = 0,1, 2,…, *n*}.

As a result, we obtain the following theorem.

**Theorem 5 .**

Problem 1|*p*_*j*_^*A*^ = *p*_*j*_ *g*(*j*, *r*), *S*_*psd*_, *CON*|*α*∑_*J*_*j*_∈*N*_*E*__ *E*_*j*_ + ∑_*J*_*j*_∈*N*_*T*__ *β*_*j*_ *U*_*j*_ + *γd* can be solved in *O*(*n* ^4^) time.

We demonstrate our approach using the following example.

**Example 6 .**

Consider the problem 1|*p*_*j*_^*A*^ = *p*_*j*_ *g*(*j*, *r*), *S*_psd_, *CON*|*α*∑_*J*_*j*_∈*N*_*E*__ *E*_*j*_ + ∑_*J*_*j*_∈*N*_*T*__ *β*_*j*_ *U*_*j*_ + *γd*.

Let *n* = 5, *h* = 3. The weights are *α* = 1, *γ* = 2, and *δ* = 0.1.

The processing times are *p*_1_ = 7, *p*_2_ = 6, *p*_3_ = 5, *p*_4_ = 2, and *p*_5_ = 1.

The tardy penalties are *β*_1_ = 10, *β*_2_ = 8, *β*_3_ = 4, *β*_4_ = 5, and *β*_5_ = 6.

The positional effects are

(16) $$\begin{array}{c} g\left(j,r\right)=\left[\begin{array}{ccccc} 2 & 1 & 3 & 2 & 4\\ 1 & 3 & 2 & 2 & 3\\ 2 & 3 & 1 & 4 & 3\\ 1 & 2 & 3 & 1 & 3\\ 2 & 1 & 2 & 3 & 4 \end{array}\right],\\ w_{1}=2.7,\;w_{2}=3.4,\;w_{3}=4. \end{array}$$

The assignment problem *A*1(3) is

(17) Min⁡ ∑j=1n∑r=1ncj,rxj,rs.t. ∑r=1nxj,r=1, j=1,2,…,n,∑j=1nxj,r=1, r=1,2,…,n,xj,r∈{0,1}, j=1,2,…,n,  r=1,2,…,n,

where

(18) cj,r=[w1g(1,1)p1w2g(1,2)p1w3g(1,3)p1β1β1w1g(2,1)p2w2g(2,2)p2w3g(2,3)p2β2β2w1g(3,1)p3w2g(3,2)p3w3g(3,3)p3β3β3w1g(4,1)p4w2g(4,2)p4w3g(4,3)p4β4β4w1g(5,1)p5w2g(5,2)p5w3g(5,3)p5β5β5]=[37.823.884101016.261.24888275120445.413.624555.43.4866].

The solution is

(19) $$\begin{array}{c} x_{j,r}=\left[\begin{array}{ccccc} 0 & 0 & 0 & 1 & 0\\ 0 & 0 & 0 & 0 & 1\\ 0 & 0 & 1 & 0 & 0\\ 1 & 0 & 0 & 0 & 0\\ 0 & 1 & 0 & 0 & 0 \end{array}\right]. \end{array}$$

The optimal sequence is π* = [*J*_4_, *J*_5_, *J*_3_, *J*_1_, *J*_2_], d* = 8.5. The total cost is 46.8.

## 4. The* SLK *Due Date Assignment Method

In the* SLK* model, *d*_*j*_ = *p*_*j*_^*A*^ + *q* (*j* = 1,2,…, *n*). We choose *γq* as the cost function *φ*(**d**), where *γ* is a positive constant. Thus, it follows from function (3) that our problem is to minimize the objective function

(20) $$\begin{array}{c} F\left(d,\pi \right)=\alpha \sum_{J_{j}\in N_{E}}E_{j}+\sum_{J_{j}\in N_{T}}\beta_{j}U_{j}+\gamma q. \end{array}$$

The problem denotes 1|*p*_*j*_^*A*^ = *p*_*j*_ *g*(*j*, *r*), *S*_psd_, *SLK*|*α*∑_*J*_*j*_∈*N*_*E*__ *E*_*j*_ + ∑_*J*_*j*_∈*N*_*T*__ *β*_*j*_ *U*_*j*_ + *γq*.

Similar to the* CON* model, if the number of jobs in *N*_*E*_ is given, we have the following solution.

**Lemma 7 .**

For the problem 1|*p*_*j*_^*A*^ = *p*_*j*_ *g*(*j*, *r*), *S*_*psd*_, *SLK*|*α*∑_*J*_*j*_∈*N*_*E*__ *E*_*j*_ + ∑_*J*_*j*_∈*N*_*T*__ *β*_*j*_ *U*_*j*_ + *γq*, if |*N*_*E*_| = *h*, there exists an optimal schedule *π* = [*J*_[1]_, *J*_[2]_,…, *J*_[*n*]_], such that the slack time *q* = *C*_[*h*−1]_ + *S*_[*h*]_.

**Proof**

The proof is similar to that of Lemma 3.

Let *π* = [*J*_[1]_, *J*_[2]_,…, *J*_[*n*]_], *q* = *C*_[*h*−1]_ + *S*_[*h*]_. Thus,

(21) q=∑j=1h−1[1+(h−j−1)δ]g([j],j)p[j] +δ∑j=1h−1g([j],j)p[j]=∑j=1h−1[1+(h−j)δ]g([j],j)p[j],E[j]=d[j]−C[j]=g([j],j)p[j]+q−C[j]=g([j],j)p[j]+∑k=1h−1[1+(h−k)δ]g([k],k)p[k] −∑k=1j[1+(h−k)δ]g([k],k)p[k]=g([j],j)p[j]+∑k=j+1h−1[1+(h−k)δ]g([k],k)p[k],1≤j≤h−1.

Consequently,

(22) ∑j=1h−1E[j]=∑j=1h−1{g([j],j)p[j]+∑k=j+1h−1[1+(h−k)δ]g([k],k)p[k]}=∑j=1h−1g([j],j)p[j] +∑j=2h−1(j−1)[1+(h−j)δ]g([j],j)p[j].

Therefore,

(23) F(d,π,h)=α∑Jj∈NEEj+∑Jj∈NTβjUj+γq=α∑j=1h−1g([j],j)p[j] +α∑j=2h−1(j−1)[1+(h−j)δ]g([j],j)p[j] +∑j=h+1nβ[j]+γq=α∑j=1h−1g([j],j)p[j] +α∑j=2h−1(j−1)[1+(h−j)δ]g([j],j)p[j] +∑j=h+1nβ[j]+γ∑j=1h−1[1+(h−j)δ]g([j],j)p[j]=∑j=1hwjg([j],j)p[j]+∑j=h+1nβ[j],

where

(24) wj={α+γ(1+(h−j)δ)for j=1,α+[α(j−1)+γ][1+δ(h−j)]for j=2,…,h−1,0for j=h.

Since the objective functions for* CON* and* SLK* due date assignment methods have the same structure, we have the following solution.

Let *x*_*j*,*r*_ be a binary variable such that *x*_*j*,*r*_ = 1 if job *J*_*j*_ is scheduled in the* r*th position and *x*_*j*,*r*_ = 0; otherwise, *j*, *r* = 1,2,…, *n*. If |*N*_*E*_| = *h*, then we can formulate the problem with objective (20) as the following assignment problem* A*2(*h*), which can be solved in *O*(*n* ^3^) time:

(25) Min⁡ ∑j=1n∑r=1ncj,rxj,rs.t.   ∑r=1nxj,r=1, j=1,2,…,n,∑j=1nxj,r=1, r=1,2,…,n,xj,r∈{0,1}, j=1,2,…,n,  r=1,2,…,n,

where

(26)  cj,r={wrg(j,r)pjfor r=1,2,…,h,βjfor r=h+1,  h+2,…,n,wr={α+γ(1+(h−r)δ)for r=1,α+[α(r−1)+γ][1+δ(h−r)]for r=2,…,h−1,0for r=h.

In order to derive the optimal solution, we have to solve the above assignment problem* A*2(*h*) for any *h* = 1,2,…, *n*.

As a result, we obtain the following theorem.

**Theorem 8 .**

The problem 1|*p*_*j*_^*A*^ = *p*_*j*_ *g*(*j*, *r*), *S*_*psd*_, *SLK*|*α*∑_*J*_*j*_∈*N*_*E*__ *E*_*j*_ + ∑_*J*_*j*_∈*N*_*T*__ *β*_*j*_ *U*_*j*_ + *γd* can be solved in *O*(*n* ^4^) time.

## 5. Job-Independent Position Effects Case

In this section, we explore the model with job-independent position effects; that is, the actual processing time of job *J*_*j*_, if scheduled in position *r* of a sequence, is given by *p*_*j*_^*A*^ = *p*_*j*_ *g*(*r*), where *g*(1), *g*(2),…, *g*(*n*) represent an array of job-independent positional factors. In Section 4, we have shown that the general version (job-dependent position effects) can be solved in *O*(*n* ^4^) time. In the following, we present an *O*(*n* ^3^) time dynamic programming algorithm for solving the special version with job-independent position effects. The main idea that will be used in the development of our algorithm is similar to that of Shabtay et al. [35].

Based on the properties proved in Section 4, we have the following solutions.

For the* CON* model, if |*N*_*E*_| = *h*, *π* = [*J*_[1]_, *J*_[2]_,…, *J*_[*n*]_], and *d* = *C*_[*h*]_, then

(27) F(d,π,h)=∑j=1h{α(j−1)+12αδ(h−j)(h+j−1)+[1+δ(h−j)]γ}g(j)p[j]+∑j=h+1nβ[j]=∑j=1hw−jg(j)p[j]+∑j=h+1nβ[j],

where

(28) w−j={α(j−1)+12αδ(h−j)(h+j−1)+[1+δ(h−j)]γ}, j=1,2,…,h.

For the* SLK* model, if |*N*_*E*_| = *h*, *π* = [*J*_[1]_, *J*_[2]_,…, *J*_[*n*]_], and *d* = *C*_[*h*−1]_ + *S*_[*h*]_, then

(29) F(d,π,h)=[α+γ(1+(h−j)δ)]g(1)p[1] +∑j=2h−1{α+[α(j−1)+γ][1+δ(h−j)]}×g(j)p[j]+∑j=h+1nβ[j]=∑j=1hw−jg(j)p[j]+∑j=h+1nβ[j],

where

(30) w−j={α+γ(1+(h−j)δ)for j=1,α+[α(j−1)+γ][1+δ(h−j)]for j=2,…,h−1,0for j=h.

Using (27) and (29), and with any of the two previously mentioned due date assignments methods, let $w_{j}={\overset{-}{w}}_{j}g(j)$; the objective function can be formulated as *F*(**d**, *π*) = ∑_*j*=1_^*n*^ *w*_*j*_ *p*_*π*(*j*)_ for the special case of *N*_*E*_ = *N*, where no jobs are tardy. From Lemma 1, the optimal job sequence is obtained by matching the largest *w*_*j*_ value to the job with the smallest *p*_*j*_ value, the second largest *w*_*j*_ value to the job with the second smallest *p*_*j*_ value, and so on. The index of the *w*_*j*_ matched with *p*_*j*_ specifies the position of job *j* in the optimal sequence. For example, first renumber the jobs in the LPT order such that *p*_1_ ≥ *p*_2_ ≥ ⋯≥*p*_*n*_, and then reorder the positional weights such that *w*_*i*_1__ ≤ *w*_*i*_2__ ≤ ⋯≤*w*_*i*_*n*__  (*i*_1_, *i*_2_,…, *i*_*n*_ is a permutation of 1,2,…, *n*), schedules job *j* in the position *i*_*j*_  (*j* = 1,2,…, *n*).

We now consider the due date assignment problem to minimize the objective function (3). Since the objective functions for all two due date assignment methods have the same structure, we provide a generic algorithm to solve these problems with two due date assignment methods. If set *N*_*E*_ is given, (|*N*_*E*_| = *h*), then we can reorder the positional weights such that *w*_*i*_1__ ≤ *w*_*i*_2__ ≤ ⋯≤*w*_*i*_*h*__. Thus, an optimal job sequence of *N*_*E*_ is obtained in *O*(*h*log⁡*h*) time. However, in order to find the optimal solution for the due date assignment problem, the contribution of the total cost of the tardy jobs must be taken into account. Below, we present a new dynamic programming algorithm. For a given *h*, the idea of a dynamic programming algorithm to minimize the function (3) is as follows. We define the states of the form (*i*, *r*), where *i* means that jobs *J*_1_, *J*_2_,…, *J*_*i*_ have been considered and *r*, (1 ≤ *r* ≤ min⁡{*i*, *h*}), represents how many of these jobs have been sequenced as nontardy jobs. A state (*i*, *r*) is associated with *f*(*i*, *r*), the smallest value of the objective function in the class of partial schedules for processing *i* jobs, provided that *r* of the these jobs has been sequenced nontardy. This method works by either each job tardy or nontardy. Next, all *f*(*i*, *r*) values can be calculated by applying the recursion for *i* = 1,2,…, *n* and *r* ≥ max⁡{1, *h* − (*n* − *i*)}. The condition is that *r* ≥ *h* − (*n* − *i*) is necessary to ensure that we do not consider states that might lead to a solution which has fewer than *r* jobs in set *N*_*E*_: since |*N*_*E*_| = *h* and there are *r* jobs that have been sequenced nontardy among the first *i* jobs, the remaining *h* − *r* nontardy jobs needed to be selected from the last *n* − *i* jobs. The formal statement of the algorithm is below.

**Algorithm 9 .**

* *

*Step 0.* Renumber the jobs in the* LPT *order such that *p*_1_ ≥ *p*_2_ ≥ ⋯≥*p*_*n*_.

*Step 1.* Calculate positional weights $w_{j}={\overset{-}{w}}_{j}g(j)$ where

(31) w−j={α(j−1)+12αδ(h−j)(h+j−1)+[1+δ(h−j)]γ}, (j=1,2,…,h)

for the* CON* model and ${\overset{-}{w}}_{1}=\alpha +\gamma [1+(h-1)\delta ]$, ${\overset{-}{w}}_{j}=\alpha +[\alpha (j-1)+\gamma ][1+\delta (h-j)]$, (*j* = 2,…, *h* − 1), and ${\overset{-}{w}}_{h}=0$ for the* SLK *model. Reorder the positional weights such that *w*_*i*_1__ ≤ *w*_*i*_2__ ≤ ⋯≤*w*_*i*_*h*__. Initialize *f*(0,0) = 0, *f*(*i*, *r*) = *∞* for *r* > *i*.

*Step 2.* For *i* from 1 to *n* calculate

(32)   f(i,0)=f(i−1,0)+βi,

(33) f(i,r)=min⁡{f(i−1,r)+βi,f(i−1,r−1)+wirpi},max⁡{1,h−(n−i)}≤r≤min⁡{i,h}.

*Step 3.* Compute the optimal value of the function f*(*h*) = *f*(*n*, *h*).

For a given *h* value, calculating all possible *f*(*n*, *h*) values using the above recursion relation requires *O*(*nh*) time. Since the value of positional weights (and the order of positional weights) can be altered by changing the *h* value, we must repeat the entire programming procedure for each *h* = 0,1, 2,…, *n*. Thus the minimal objective value, F*, is given by

(34) $$\begin{array}{c} F^{*}=\underset{h=0,1,\ldots ,n}{\min}\left\{f^{*}\left(h\right)\right\}. \end{array}$$

Therefore, the following statement holds.

**Theorem 10 .**

Both the problems 1|*p*_*j*_^*A*^ = *p*_*j*_ *g*(*r*), *S*_*psd*_, *CON*|*α*∑_*J*_*j*_∈*N*_*E*__ *E*_*j*_ + ∑_*J*_*j*_∈*N*_*T*__ *β*_*j*_ *U*_*j*_ + *γd* and 1|*p*_*j*_^*A*^ = *p*_*j*_ *g*(*r*), *S*_*psd*_, *SLK*|*α*∑_*J*_*j*_∈*N*_*E*__ *E*_*j*_ + ∑_*J*_*j*_∈*N*_*T*__ *β*_*j*_ *U*_*j*_ + *γd* can be solved in *O*(*n* ^3^) time.

**Example 11 .**

Consider the problem 1|*p*_*j*_^*A*^ = *p*_*j*_ *g*(*r*), *S*_psd_, *CON*|*α*∑_*J*_*j*_∈*N*_*E*__ *E*_*j*_ + ∑_*J*_*j*_∈*N*_*T*__ *β*_*j*_ *U*_*j*_ + *γd*.

Let *n* = 5 and *h* = 3. The weights are *α* = 1, *γ* = 2, and *δ* = 0.1.

The processing times are *p*_1_ = 7, *p*_2_ = 6, *p*_3_ = 5, *p*_4_ = 2, and *p*_5_ = 1.

The tardy penalties are *β*_1_ = 10, *β*_2_ = 8, *β*_3_ = 4, *β*_4_ = 5, and *β*_5_ = 6.

The positional effects are *g*(1) = 8, *g*(2) = 7, *g*(3) = 3, *g*(4) = 4, and *g*(5) = 7.

Positional weights are *w*_1_ = 21.6, *w*_2_ = 23.8, and *w*_3_ = 12.

Positional weights are reordered such that *w*_*i*_1__ ≤ *w*_*i*_2__ ≤ *w*_*i*_3__; that is, *w*_*i*_1__ = *w*_3_, *w*_*i*_2__ = *w*_1_, and *w*_*i*_3__ = *w*_2_:

(35) f(0,0)=0,i=1,f(1,0)=f(0,0)+β1=10,f(1,1)=f(0,0)+wi1p1=84,i=2,f(2,0)=f(1,0)+β2=18,f(2,1)=min⁡{f(1,1)+β2,f(1,0)+wi1p2}=min⁡{92,82}=82,f(2,2)=f(1,1)+wi2p2=203.6,i=3,f(3,0)=f(2,0)+β3=22,f(3,1)=min⁡{f(2,1)+β3,f(2,0)+wi1p3}=min⁡{86,78}=78,f(3,2)=min⁡{f(2,2)+β3,f(2,1)+wi2p3}=min⁡{207.6,190}=190,f(3,3)=f(2,2)+wi3p3=322.6,i=4,f(4,0)=f(3,0)+β4=27,f(4,2)=min⁡{f(3,2)+β4,f(3,1)+wi2p4}=min⁡{195,121.2}=121.2,f(4,3)=min⁡{f(3,3)+β4,f(3,2)+wi3p4}=min⁡{327.6,237.6}=237.6,i=5,f(5,0)=f(4,0)+β5=33,f(5,3)=min⁡{f(4,3)+β5,f(4,2)+wi3p5}=min⁡{243.6,145}=145.

Therefore, *f*(3) = *f*(5,3) = 145.

The optimal sequence is π* = [*J*_4_, *J*_5_, *J*_3_, *J*_1_, *J*_2_], d* = 41.9. The total cost is 145.

## 6. Conclusions

Scheduling problems involving position-dependent processing time have received increasing attention in recent years. In this paper, we considered single machine scheduling and due date assignment with setup time in which a job's processing time depends on its position in a sequence. The setup time is past-sequence-dependent (p-s-d). The objective functions include total earliness, the weighted number of tardy jobs, and the cost of due date assignment. The due date assignment methods used in this problem include common due date (*CON*) and equal slack (*SLK*). We have presented an *O*(*n* ^4^) time algorithm for the general case and an *O*(*n* ^3^) time dynamic programming algorithm for the special cases. In the paper, the model with position-dependent effects is considered. However, in some other situations, a job's processing time may be time-dependent or both position-dependent and time-dependent. Therefore, it is worthwhile for future research to investigate the model in which a job's processing time depends both on its position in a sequence and its start time. It is also interesting for future research to investigate the model in the context of other scheduling settings, including multimachine and job-shop scheduling.
